# 3-Chloro-*N*-phenyl­benzamide

**DOI:** 10.1107/S1600536811047805

**Published:** 2011-11-16

**Authors:** Vinola Z. Rodrigues, Peter Herich, B. Thimme Gowda, Jozef Kožíšek

**Affiliations:** a Department of Chemistry, Mangalore University, Mangalagangotri 574 199, Mangalore, India; b Institute of Physical Chemistry and Chemical Physics, Slovak University of Technology, Radlinského 9, SK-812 37 Bratislava, Slovak Republic

## Abstract

In the title compound, C_13_H_10_ClNO, the *meta*-chloro group on the benzoyl ring is positioned *syn* to the C=O bond. The two aromatic rings make a dihedral angle of 88.5 (3)°. In the crystal, N—H⋯O hydrogen bonds link the mol­ecules into *C*(4) chains  propagating in [010].

## Related literature

For the preparation of the title compound, see: Gowda *et al.* (2003 ▶). For our studies on the effects of substituents on the structures and other aspects of *N*-(ar­yl)-amides, see: Bhat & Gowda (2000 ▶); Bowes *et al.* (2003 ▶); Gowda *et al.* (2008 ▶); Saeed *et al.* (2010 ▶), on *N*-(ar­yl)-methane­sulfonamides, see: Gowda *et al.* (2007 ▶), on *N*-(ar­yl)-aryl­sulfonamides, see: Shetty & Gowda (2005 ▶) and on *N*-chloro-amides, see: Gowda & Weiss (1994 ▶).
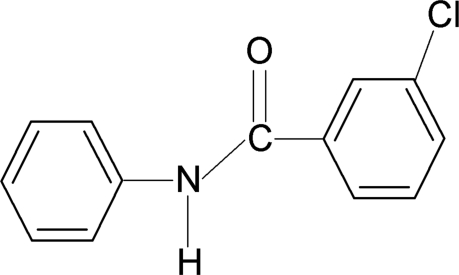


## Experimental

### 

#### Crystal data

C_13_H_10_ClNO
*M* *_r_* = 231.67
Monoclinic, 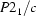

*a* = 25.0232 (9) Å
*b* = 5.3705 (2) Å
*c* = 8.1289 (3) Å
β = 98.537 (3)°
*V* = 1080.32 (7) Å^3^
*Z* = 4
Mo *K*α radiation
μ = 0.33 mm^−1^
*T* = 293 K
0.90 × 0.79 × 0.05 mm

#### Data collection

Oxford Xcalibur Ruby Gemini diffractometer
Absorption correction: analytical [*CrysAlis RED* (Oxford Diffraction, 2009 ▶), based on expressions derived by Clark & Reid (1995 ▶)] *T*_min_ = 0.752, *T*_max_ = 0.984
18170 measured reflections
3020 independent reflections
2270 reflections with *I* > 2σ(*I*)
*R*_int_ = 0.024

#### Refinement

*R*[*F* ^2^ > 2σ(*F* ^2^)] = 0.043
*wR*(*F* ^2^) = 0.124
*S* = 1.03
3020 reflections
145 parameters
H-atom parameters constrained
Δρ_max_ = 0.48 e Å^−3^
Δρ_min_ = −0.34 e Å^−3^

### 

Data collection: *CrysAlis CCD* (Oxford Diffraction, 2009 ▶); cell refinement: *CrysAlis CCD*; data reduction: *CrysAlis RED* (Oxford Diffraction, 2009 ▶); program(s) used to solve structure: *SHELXS97* (Sheldrick, 2008 ▶); program(s) used to refine structure: *SHELXL97* (Sheldrick, 2008 ▶); molecular graphics: *DIAMOND* (Brandenburg, 2002 ▶); software used to prepare material for publication: *enCIFer* (Allen *et al.*, 2004 ▶).

## Supplementary Material

Crystal structure: contains datablock(s) I, global. DOI: 10.1107/S1600536811047805/ds2154sup1.cif

Structure factors: contains datablock(s) I. DOI: 10.1107/S1600536811047805/ds2154Isup2.hkl

Supplementary material file. DOI: 10.1107/S1600536811047805/ds2154Isup3.cml

Additional supplementary materials:  crystallographic information; 3D view; checkCIF report

## Figures and Tables

**Table 1:** Hydrogen-bond geometry (Å, °)

*D*—H⋯*A*	*D*—H	H⋯*A*	*D*⋯*A*	*D*—H⋯*A*
N1—H1*A*⋯O1^i^	0.86	2.40	3.2377 (17)	165

Symmetry code: (i) 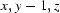.

**Fig. 1.**
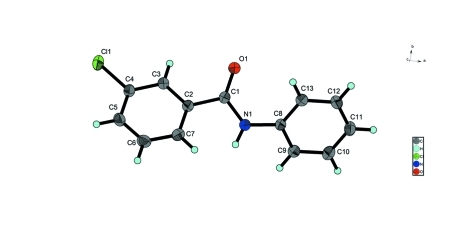
Molecular structure of the title compound showing the atom labelling scheme. Displacement ellipsoids are drawn at the 50% probability level. H atoms are represented as small spheres of arbitrary radii.

**Fig. 2.**
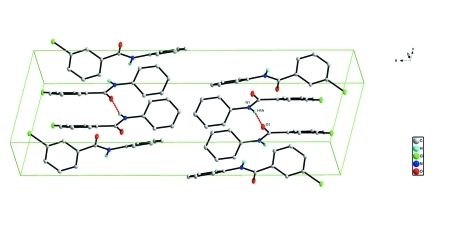
Part of the crystal structure of the title compound. Molecular chains are generated by N—H···O hydrogen bonds which are shown by dashed lines.

## supplementary crystallographic information

### Comment

The amide and sulfonamide moieties are the constituents of many biologically significant compounds. As part of our studies on the substituent effects on the structures and other aspects of *N*-(aryl)-amides (Bhat & Gowda, 2000; Bowes *et al.*, 2003; Gowda *et al.*, 2008; Saeed *et al.*, 2010, *N*-(aryl)-methanesulfonamides (Gowda *et al.*, 2007), *N*-(aryl)-arylsulfonamides (Shetty & Gowda, 2005) and *N*-chloro-arylamides (Gowda & Weiss, 1994), in the present work, the crystal structure of 3-Chloro-*N*-(phenyl)benzamide (I) has been determined (Fig.1).

In (I), the *meta*-chloro group in the benzoyl ring is positioned *syn* to the C=O bond, while the N—H and C=O bonds in the C—NH—C(O)—C segment are *anti* to each other, similar to that observed in 3-Chloro-*N*-(3-chlorophenyl)-benzamide (II) (Gowda *et al.*, 2008). Further, the two aromatic rings in (I) make the dihedral angle of 88.5 (3)°.

In the crystal structure, intermolecular N—H···O hydrogen bonds link the molecules into infinite chains running along the *c*-axis. Part of the crystal structure is shown in Fig. 2.

### Experimental

The title compound was prepared according to the method described by Gowda *et al.* (2003). The purity of the compound was checked by determining its melting point. It was characterized by recording its infrared and NMR spectra.

Rod like colorless single crystals of the title compound used in X-ray diffraction studies were obtained by slow evaporation of an ethanol solution of the compound (0.5 g in about 30 ml of ethanol) at room temperature.

### Refinement

All H atoms were visible in difference maps and then treated as riding atoms with C–H distances of 0.93Å (C-aromatic) and N—H = 0.86 Å. The *U*_iso_(H) values were set at 1.2 *U*_eq_(C-aromatic, N).

### Crystal data

**Table:**

C_13_H_10_ClNO	*F*(000) = 480
*M**_r_* = 231.67	*D*_x_ = 1.424 Mg m^−^^3^
Monoclinic, *P*2_1_/*c*	Mo *K*α radiation, λ = 0.71073 Å
Hall symbol: -P 2ybc	Cell parameters from 6202 reflections
*a* = 25.0232 (9) Å	θ = 3.8–29.5°
*b* = 5.3705 (2) Å	µ = 0.33 mm^−^^1^
*c* = 8.1289 (3) Å	*T* = 293 K
β = 98.537 (3)°	Rod, colorless
*V* = 1080.32 (7) Å^3^	0.90 × 0.79 × 0.05 mm
*Z* = 4	

### Data collection

**Table:**

Oxford Xcalibur Ruby Gemini diffractometer	3020 independent reflections
Radiation source: Enhance (Mo) X-ray Source	2270 reflections with *I* > 2σ(*I*)
graphite	*R*_int_ = 0.024
Detector resolution: 10.4340 pixels mm^-1^	θ_max_ = 29.5°, θ_min_ = 3.8°
ω scans	*h* = −34→34
Absorption correction: analytical [*CrysAlis RED* (Oxford Diffraction, 2009), based on expressions derived by Clark & Reid (1995)]	*k* = −7→7
*T*_min_ = 0.752, *T*_max_ = 0.984	*l* = −11→11
18170 measured reflections	

### Refinement

**Table:**

Refinement on *F*^2^	Primary atom site location: structure-invariant direct methods
Least-squares matrix: full	Secondary atom site location: difference Fourier map
*R*[*F*^2^ > 2σ(*F*^2^)] = 0.043	Hydrogen site location: inferred from neighbouring sites
*wR*(*F*^2^) = 0.124	H-atom parameters constrained
*S* = 1.03	*w* = 1/[σ^2^(*F*_o_^2^) + (0.063*P*)^2^ + 0.3141*P*] where *P* = (*F*_o_^2^ + 2*F*_c_^2^)/3
3020 reflections	(Δ/σ)_max_ < 0.001
145 parameters	Δρ_max_ = 0.48 e Å^−^^3^
0 restraints	Δρ_min_ = −0.34 e Å^−^^3^

### Special details

**Table:**

Experimental. CrysAlis RED (Oxford Diffraction, 2009) Analytical numeric absorption correction using a multifaceted crystal model based on expressions derived (Clark & Reid, 1995).
Geometry. All e.s.d.'s (except the e.s.d. in the dihedral angle between two l.s. planes) are estimated using the full covariance matrix. The cell e.s.d.'s are taken into account individually in the estimation of e.s.d.'s in distances, angles and torsion angles; correlations between e.s.d.'s in cell parameters are only used when they are defined by crystal symmetry. An approximate (isotropic) treatment of cell e.s.d.'s is used for estimating e.s.d.'s involving l.s. planes.
Refinement. Refinement of *F*^2^ against ALL reflections. The weighted *R*-factor *wR* and goodness of fit *S* are based on *F*^2^, conventional *R*-factors *R* are based on *F*, with *F* set to zero for negative *F*^2^. The threshold expression of *F*^2^ > σ(*F*^2^) is used only for calculating *R*-factors(gt) *etc*. and is not relevant to the choice of reflections for refinement. *R*-factors based on *F*^2^ are statistically about twice as large as those based on *F*, and *R*- factors based on ALL data will be even larger.

### Fractional atomic coordinates and isotropic or equivalent isotropic displacement parameters (Å^2^)

**Table:**

	*x*	*y*	*z*	*U*_iso_*/*U*_eq_	
C1	0.24828 (6)	0.1506 (3)	0.40582 (19)	0.0408 (3)	
C2	0.19382 (5)	0.0662 (3)	0.44101 (18)	0.0387 (3)	
C3	0.14947 (6)	0.2149 (3)	0.38313 (19)	0.0408 (3)	
H3A	0.1539	0.3624	0.3268	0.049*	
C4	0.09844 (6)	0.1399 (3)	0.41060 (19)	0.0421 (3)	
C5	0.09099 (6)	−0.0774 (3)	0.4947 (2)	0.0474 (4)	
H5A	0.0565	−0.1266	0.5109	0.057*	
C6	0.13523 (7)	−0.2200 (3)	0.5540 (2)	0.0505 (4)	
H6A	0.1306	−0.3650	0.6129	0.061*	
C7	0.18657 (6)	−0.1510 (3)	0.5275 (2)	0.0451 (3)	
H7A	0.2161	−0.2500	0.5675	0.054*	
C8	0.33745 (5)	−0.0128 (3)	0.35872 (18)	0.0374 (3)	
C9	0.35764 (6)	−0.1981 (3)	0.2665 (2)	0.0445 (3)	
H9A	0.3354	−0.3283	0.2231	0.053*	
C10	0.41083 (7)	−0.1893 (3)	0.2391 (2)	0.0511 (4)	
H10A	0.4242	−0.3134	0.1767	0.061*	
C11	0.44399 (6)	0.0017 (3)	0.3037 (2)	0.0505 (4)	
H11A	0.4798	0.0069	0.2853	0.061*	
C12	0.42390 (6)	0.1857 (3)	0.3960 (2)	0.0497 (4)	
H12A	0.4464	0.3147	0.4400	0.060*	
C13	0.37059 (6)	0.1805 (3)	0.4241 (2)	0.0451 (4)	
H13A	0.3573	0.3053	0.4862	0.054*	
Cl1	0.042556 (15)	0.32323 (9)	0.33863 (6)	0.06042 (18)	
N1	0.28327 (5)	−0.0372 (2)	0.38904 (16)	0.0421 (3)	
H1A	0.2714	−0.1863	0.3976	0.051*	
O1	0.25904 (5)	0.3707 (2)	0.39138 (18)	0.0587 (3)	

### Atomic displacement parameters (Å^2^)

**Table:**

	*U*^11^	*U*^22^	*U*^33^	*U*^12^	*U*^13^	*U*^23^
C1	0.0323 (7)	0.0365 (8)	0.0530 (8)	0.0019 (5)	0.0046 (6)	0.0003 (6)
C2	0.0340 (7)	0.0361 (7)	0.0465 (7)	0.0002 (5)	0.0074 (6)	−0.0040 (6)
C3	0.0342 (7)	0.0363 (7)	0.0528 (8)	−0.0005 (5)	0.0088 (6)	−0.0001 (6)
C4	0.0328 (7)	0.0417 (8)	0.0522 (8)	−0.0004 (6)	0.0079 (6)	−0.0051 (6)
C5	0.0405 (8)	0.0457 (9)	0.0589 (9)	−0.0068 (6)	0.0174 (7)	−0.0050 (7)
C6	0.0564 (10)	0.0402 (8)	0.0584 (10)	−0.0030 (7)	0.0199 (8)	0.0050 (7)
C7	0.0439 (8)	0.0399 (8)	0.0521 (8)	0.0052 (6)	0.0096 (6)	0.0025 (6)
C8	0.0306 (6)	0.0353 (7)	0.0461 (7)	0.0018 (5)	0.0047 (5)	0.0036 (6)
C9	0.0384 (8)	0.0397 (8)	0.0549 (9)	0.0003 (6)	0.0053 (6)	−0.0042 (6)
C10	0.0427 (8)	0.0525 (10)	0.0600 (10)	0.0074 (7)	0.0133 (7)	−0.0040 (7)
C11	0.0342 (7)	0.0553 (10)	0.0634 (10)	0.0011 (7)	0.0118 (7)	0.0063 (8)
C12	0.0375 (8)	0.0454 (9)	0.0650 (10)	−0.0074 (6)	0.0035 (7)	0.0014 (7)
C13	0.0383 (7)	0.0392 (8)	0.0573 (9)	−0.0003 (6)	0.0059 (6)	−0.0043 (6)
Cl1	0.0316 (2)	0.0611 (3)	0.0886 (4)	0.00427 (16)	0.00877 (19)	0.0092 (2)
N1	0.0326 (6)	0.0342 (6)	0.0599 (7)	−0.0004 (5)	0.0085 (5)	−0.0018 (5)
O1	0.0390 (6)	0.0354 (6)	0.1034 (10)	0.0009 (4)	0.0155 (6)	0.0043 (6)

### Geometric parameters (Å, °)

**Table:**

C1—O1	1.2220 (18)	C8—C13	1.384 (2)
C1—N1	1.3558 (18)	C8—C9	1.385 (2)
C1—C2	1.5033 (19)	C8—N1	1.4195 (18)
C2—C7	1.387 (2)	C9—C10	1.382 (2)
C2—C3	1.391 (2)	C9—H9A	0.9300
C3—C4	1.3881 (19)	C10—C11	1.374 (2)
C3—H3A	0.9300	C10—H10A	0.9300
C4—C5	1.379 (2)	C11—C12	1.380 (2)
C4—Cl1	1.7390 (15)	C11—H11A	0.9300
C5—C6	1.374 (2)	C12—C13	1.387 (2)
C5—H5A	0.9300	C12—H12A	0.9300
C6—C7	1.384 (2)	C13—H13A	0.9300
C6—H6A	0.9300	N1—H1A	0.8600
C7—H7A	0.9300		
			
O1—C1—N1	123.72 (14)	C13—C8—C9	120.04 (13)
O1—C1—C2	121.93 (13)	C13—C8—N1	122.38 (13)
N1—C1—C2	114.34 (12)	C9—C8—N1	117.51 (13)
C7—C2—C3	119.74 (13)	C10—C9—C8	119.99 (15)
C7—C2—C1	122.71 (13)	C10—C9—H9A	120.0
C3—C2—C1	117.55 (13)	C8—C9—H9A	120.0
C4—C3—C2	119.06 (14)	C11—C10—C9	120.34 (15)
C4—C3—H3A	120.5	C11—C10—H10A	119.8
C2—C3—H3A	120.5	C9—C10—H10A	119.8
C5—C4—C3	121.36 (14)	C10—C11—C12	119.62 (15)
C5—C4—Cl1	118.99 (11)	C10—C11—H11A	120.2
C3—C4—Cl1	119.65 (12)	C12—C11—H11A	120.2
C6—C5—C4	118.99 (14)	C11—C12—C13	120.81 (15)
C6—C5—H5A	120.5	C11—C12—H12A	119.6
C4—C5—H5A	120.5	C13—C12—H12A	119.6
C5—C6—C7	120.94 (15)	C8—C13—C12	119.20 (14)
C5—C6—H6A	119.5	C8—C13—H13A	120.4
C7—C6—H6A	119.5	C12—C13—H13A	120.4
C6—C7—C2	119.88 (15)	C1—N1—C8	126.62 (13)
C6—C7—H7A	120.1	C1—N1—H1A	116.7
C2—C7—H7A	120.1	C8—N1—H1A	116.7
			
O1—C1—C2—C7	−150.79 (16)	C1—C2—C7—C6	−179.20 (15)
N1—C1—C2—C7	30.3 (2)	C13—C8—C9—C10	−0.3 (2)
O1—C1—C2—C3	29.3 (2)	N1—C8—C9—C10	−177.28 (14)
N1—C1—C2—C3	−149.65 (14)	C8—C9—C10—C11	0.4 (3)
C7—C2—C3—C4	−1.2 (2)	C9—C10—C11—C12	−0.2 (3)
C1—C2—C3—C4	178.75 (13)	C10—C11—C12—C13	−0.1 (3)
C2—C3—C4—C5	0.4 (2)	C9—C8—C13—C12	0.0 (2)
C2—C3—C4—Cl1	−179.84 (11)	N1—C8—C13—C12	176.84 (14)
C3—C4—C5—C6	0.9 (2)	C11—C12—C13—C8	0.2 (3)
Cl1—C4—C5—C6	−178.89 (13)	O1—C1—N1—C8	2.4 (3)
C4—C5—C6—C7	−1.3 (3)	C2—C1—N1—C8	−178.75 (13)
C5—C6—C7—C2	0.5 (3)	C13—C8—N1—C1	34.8 (2)
C3—C2—C7—C6	0.7 (2)	C9—C8—N1—C1	−148.36 (15)

### Hydrogen-bond geometry (Å, °)

**Table:**

*D*—H···*A*	*D*—H	H···*A*	*D*···*A*	*D*—H···*A*
N1—H1A···O1^i^	0.86	2.40	3.2377 (17)	165.

Symmetry codes: (i) *x*, *y*−1, *z*.
